# Anti-diabetic activity of field cricket glycosaminoglycan by ameliorating oxidative stress

**DOI:** 10.1186/s12906-020-03027-x

**Published:** 2020-07-22

**Authors:** Mi Young Ahn, Ban Ji Kim, Ha Jeong Kim, Jang Mi Jin, Hyung Joo Yoon, Jae Sam Hwang, Byung Mu Lee

**Affiliations:** 1 Department of Agricultural Biology, National Institute of Agricultural Sciences, RDA, 166 Nongsaengmyung-Ro, Iseo-Myun, Wanju-Gun, 55365 South Korea; 2 Korean Basic Science Institute, Ochang, 863-883 South Korea; 3 Division of Toxicology, College of Pharmacy, Sungkyunkwan University, Chunchun-dong 300, Changan-ku, Gyeonggi-do, Suwon, 440-746 South Korea

**Keywords:** Cricket glycosaminoglycan, N-glycan, Homo db mice, Anti-oxidant enzyme

## Abstract

**Background:**

Field cricket (*Gryllus bimaculatus*) is newly emerged as an edible insect in several countries. Anti-inflammatory effect of glycosaminoglycan derived from this cricket on chronic disease animal model such as diabetic mouse has not been fully investigated yet. Thus, the objective of this study was to determine the anti-oxidative effect of such glycosaminoglycan on diabetic mouse.

**Methods:**

To discover potential therapeutic agents, field cricket glycosaminoglycan (GbG) was tested in the present study. Its anti-oxidative activities in diabetic mice were determined based on its abilities to reduce glucose, ALT, AST, ALP, LDL-cholesterol and BUN levels. Dung beetle (*C. molossus*) glycosaminoglycan (CaG) was used as a positive control. Db mice were intraperitoneally administered for 1 month according to their group assignments: 1) normal (DB-Hetero); 2) control (DB-Homo); 3) 5 mg/kg treatment of CaG (CaG5); 4) 5 mg/kg treatment of GbG (GbG5); and 5) 10 mg/kg treatment of metformin (Metformin 10).

**Results:**

Blood glucose level decreased after 1st week of treatment with GbG. LDL-cholesterol and alkaline phosphatase levels were also inhibited by GbG. Markers of oxidative damage, such as protein carbonyl content and levels of hepatocellular biomarkers, were reduced in db mice treated with GbG. Especially anti-oxidative activities of catalase, superoxide dismutase and glutathione peroxidase were significantly increased in GbG treated group compared to those in the control (Db Homo). GbG was composed of heparin disaccharides. Its main N-glycan was identified as Hex_9_GlcNAc_2_ (m/z 1905.7) with neutral mono-sugar mainly comprising of hexose and L (+) rhamnose by mass spectroscopy.

**Conclusions:**

Sero-biochemical and hepatocellular anti-oxidant assay results in db mice suggest that cricket (*G. bimaculatus*) glycosaminoglycan might possess anti-oxidative effect in diabetic state.

## Background

Eating insects is becoming common worldwide. Edible crickets acquired from industrial rearing systems as alternative food sources are increasingly used in several countries including the Netherlands. *Gryllus bimaculatus* (field cricket, Gb) water extract has been used in Oriental medicine as a crude antifebrile drug and a high blood pressure lowering agent. Recently, it has been shown that Gb water extract can lower blood ethanol metabolite concentrations by enhancing levels of liver mitochondrial alcohol and acetaldehyde dehydrogenases [1]. It also possesses anti-obesity effect by inhibiting adipose tissue accumulation in high-fat-diet induced diabetic rats [2]. We have recently shown that consumption of edible crickets has significant beneficial effects on gut microbiota in healthy adults [3].

Type-2 diabetes is known to be associated with oxidative stress. It influences vascular chronic diseases such as atherosclerosis, hypertension and nephropathy [4]. Antioxidant and anti-inflammatory activities of enzymatic hydrolysates and peptide fractions from selected heat-treated edible insects including cricket have been ascertained [5] except for other carbohydrate components of field cricket. Indeed, all human cells are coated with an array of glycoproteins, glycolipids and polysaccharides named glycocalyx, the surface proteoglycan/glycoprotein layer [6]. Therefore, glycans can appeal distinct properties as biomarker targets [7]. Especially, N-glycans are ubiquitous in nature. They provide structural and functional stability to N-linked glycoprotein, with flexibility [8].

In the overview of a total insect component survey concept, as one of the functional components, Glycosaminoglycan (GAG), a mucin polysaccharide as one of functional components of edible insects, needs to be standardized and manufactured from a routine (no versatile) natural source with the same insect rearing conditions. The distinctive role of Gb GAG has been reported to possess anti-inflammatory effect in arthritis induced rat model [9]. It also has anti-obesity [10] and antilipidemic [11] effects in high-fat-diet rats. Some GAGs have robust anti-oxidant activity that they can scavenge free radicals and repair cellular oxidative damages [12].

Db/db mouse model is commonly used to conduct research on type 2 diabetes mellitus (DM) and its comorbidities including obesity and hypertension [13]. Some comparative studies have shown heparin sulfate to be a type of glycosaminoglycan plays a crucial role in proliferation, development and maturation of β cells, thereby contributing to normal glucose hemostasis [14] and mouse β cell survival in autoimmune type 1 diabetes mellitus [15]. However, the anti-inflammatory effect of glycosaminoglycan derived from this cricket on chronic disease animal model such as diabetic mouse has not been fully investigated yet. In the present study, the anti-oxidant activities of Gb GAG contributed to its anti-diabetic activity. Attenuation of the complex metabolic depression in diabetes may be mediated via low levels of anti-oxidative enzyme, which repaired the cellular oxidative damage. Protein carbonyl level and induction of antioxidant enzyme ratios following GAG treatment of db/db mice were also analyzed. Dung beetle (*Catharsius molossus*, Ca) GAG known to exhibit marked anti-ageing activity characterized by reduced oxidative damage in aged rats [16] was used as a positive control. In this work, we have also characterized purified N-glycans for deglycosylation of GbGAG followed by MS and MS/MS (MALDI TOF MS) analysis. In this study, we found that field cricket carried elements expressing glycosaminoglycan with anti-oxidant activities to ameliorate cellular oxidative damage in diabetes.

## Methods

### Preparation of field cricket glycosaminoglycan

Field crickets (*G. bimaculatus*) were obtained from a cricket farm located in Hwasung, Korea. They were freeze-dried in NAAS (National Academy of Agricultural Science), South Korea. Dried dung beetles (*C. molossus*) were purchased from a local market in China. They were prepared by Chinese phamacognosists and posted to NAAS in 2002 (no law regulation time). These insects were defatted, freezing dried, and stored in a deep freezer.

Each insect glycosaminoglycan was purified with a method as reported previously [17], involving removal of fat with ethanol and acetone, protein enzymatic hydrolysis by treatment with alcalase (Sigma Aldrich, USA), protein precipitation with trichloroacetic acid (5%), impure materials cleansing with detergent such as cetylpyridinium chloride (5%), and dissolving of non-glycosaminoglycan with 2.5 M NaCl. GAGs were then acquired through cold ethanol precipitation, centrifugation, and DEAE Sephadex A-25 gel chromatography. For structural identification of these GAGs, main peaks of digested Gb GAG after co-incubation with heparinase I, II, or III were pooled using SAX column (Phenomenex, USA) with high performance liquid chromatography (HPLC) to determine its purity. They were compared with heparin disaccharides or carbohydrate standards with an ESI Mass Spectrometer (SYNAPT G2, Waters, U.K.).

### N-glycan preparation derived from cricket glycosaminoglycan

A purified cricket glycosaminoglycan through DEAE Sephadex A-25 gel from 0.5 M NaCl fraction (GbG 0.5 M) was further purified to obtain a low molecular weight. Using a previously reported method [18], this GbG was denatured by boiling for 10 min. It was then incubated with 1000 U of peptide N-glycosidase F, PNGase F (New England biolabs, USA) to release N-glycan. This cricket N-glycan was then solid-phase extracted using Graphitized carbon cartridges (extract clean™ carbo, Grace davision discovery Sciences, USA). N-glycan derived from GbG was then characterized by liquid chromatography for mass and mass/mass analyses with a MADI-TOF analyzer (AXIMA Resonance, Shimadzu).

### Neutral mono-sugar composition of digested N-glycan on the basis of GC-MS analysis

Isolated Gb N-glycan (100 μL) was HCl-hydrolyzed, trimethylsilylated using *N*, *O*-Bis(trimethylsilyl)trifluoroacetamide containing 1% trimethylchlorosilane (Sigma Co., USA) and then injected into a GC-MS (Agilent, USA) [11].

### Animals

Two kinds of db mice, BKS.Cg-m+/+*Lepr*^db^, heterozygous (db/+) (DB-Hetero, normal) and homozygotes (db/db) (DB-Homo, diabetes) male db mice at 12 weeks of age, were purchased from Samtako Co. Ltd. (Osan, Korea). All experiments were performed in accordance with NIH Guidelines for Care and Use of laboratory Animals. Experimental protocols were approved by Laboratory Animals’ Ethical committee of National Institute of Agricultural Sciences, Republic of Korea (approval number: NIAS201605).

These mice were allocated into two control groups (negative and positive controls) and two GAG treatment groups (11 mice per group). They were distributed according to their similarity in weight (27.86 ± 1.14 g for DB-Hetero, 46.73 ± 4.73 g for DB-Homo). Treatments were intraperitoneally offered in phosphate buffered saline daily under normal diet (AIN-76A rodent diet, Research Diet). The following treatment groups were used: 1) normal (DB-Hetero); 2) control (DB-Homo); 3) 5 mg/kg treatment of CaG (CaG5); 4) 5 mg/kg treatment of GbG (GbG5); and 5) 10 mg/kg treatment of metformin (Metformin 10) as positive control. After overnight fast, euthanasia was performed under light CO_2_ inhalation at the end of one-month treatment. Blood samples were collected from abdominal aorta with a syringe. Organs of mice were extracted via autopsy.

### Body weight and estimation of blood glucose levels

Body weights were weekly determined. Non-fasting glucose levels in blood samples drawn from tail veins were determined weekly using a blood glucose Nocodingone detector (Theragenetex.Co., Sungnam, Korea). On the last day of the one-month treatment schedule in a fasted state, serum glucose levels were determined with an auto-analyzer and compared with those of mice treated with metformin, a positive control drug for type 2 diabetes [19].

### Organ and adipose fat weights

Absolute weights of adrenal glands, kidneys, heart, liver, lung, spleen, stomach, and pancreas were measured. Their relative weights (organ-to-body ratio) were then calculated. Abdominal (including omental and perirenal) and epididymal fat to-body weight ratios of each group were also determined and compared with those of the DB-Homo control group.

### Blood sampling and sero-parameter assay

After treatment with CaG5, GbG5, or metformin10 for 1 month, all non-diabetic control, db control, and db treated mice were blood sampled and evaluated for the following parameters with an auto analyzer (Hitachi 7060 Automatic Clinical Analyzer, Tokyo, Japan) according to Green Cross Lab’s manual: albumin, hyaluronic acid, free fatty acid, ALP, AST, ALT, lactic dehydrogenase, creatinine phosphokinase, total cholesterol, triglyceride, HDL cholesterol, LDL cholesterol, creatinine, BUN, total protein, Na, Cl, c-reactive protein, Ca, K, total IgE, C-peptide, and insulin.

### Carbonyl content detection for oxidative protein damage

Protein oxidative stress was evaluated by measuring protein carbonyl contents in the supernatant of liver homogenate and blood after centrifugation using an OxiSelect™ protein carbonyl ELISA kit (Cell Biolabs, Inc., San Diego, CA, USA).

### Measurements of oxidative enzyme activities

Supernatant of each liver homogenate after centrifugation was obtained to determine catalase, glutathione peroxidase, glutathione s-transferase, and superoxidase dismutase activities using an OxiSelect™ ELISA kit as described previously [12, 20, 21].

### Endothelial nitric oxide synthase, laminin and VEGF on diabetic endothelial cells

Endothelial vasorelaxation related to *e*NOS and growth factor were also measured in diabetic type 2 microvascular endothelial cells (D- HMVECs) (Clonetics™, diabetic type II, Lonza CC-2928, Cambrex, Walkersville, USA). D-HMVECs were pretreated with 0.2 mg/ml of either GAG or Pravastatin and incubated prior to determination of *e*NOS [22] and VEGF [23] (Quantikine, R&D Systems, Inc., Minneapolis, USA). Laminin level in diabetic HMVEC cells was measured using a Quantimatrix™ human laminin ELISA kit (Millipore, Billerica, USA). Positive controls were Pravastatin (CJ Healthcare Co., Seoul, Korea) and chitosan (Sigma Co., USA).

### Adipocyte density and pathological observation

Excised organs including kidneys, heart, liver, lung, spleen, stomach, pancreas, testis, and adipose tissues of db mice were fixed with 10% neutral formalin. After paraffin molding, their slides were stained with hematoxylin and eosin and toluidine blue O, examined with a light microscope (Leica CTR6000, Hesse, Germany), and photographed. Toluidine blue O stained adipocyte densities (cells/mm^2^) in treated and control tissues were determined by counting (original magnification, × 400).

### Statistical analysis

Analysis of variance (ANOVA) was performed to determine differences in means and standard errors of parameters of different groups. To determine significant differences between control and treated groups, Student’s *t*-test was performed. Same groups were subjected to repeated measures ANOVA at different time points followed by pairwise multiple comparisons (Tukey’s test).

## Results

### Field cricket glycosaminoglycan preparation

From 1 kg of each dried insect, the yield of freeze-dried GAG powder was about 1.52 g for CaG and 3.4 g for GbG.

### Analysis of field cricket glycosaminoglycan

**Fig. 1:**
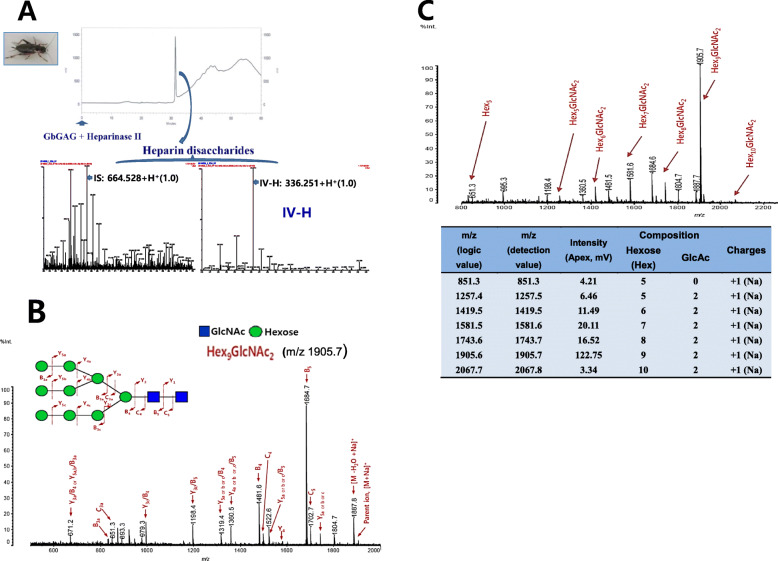
Identification of cricket glycosaminoglycan. **a** Field cricket (*G. bimaculatus*) glycosaminoglycan degradation: HPLC chromatogram of GbG that digested by heparinase II and ESI Mass Spectrometer chromatogram (Time of flight analyzer). **b** N-glycan identification: N-glycan from *G. bimaculatus* glycosaminoglycan using MALDI MS/MS TOF analyzer (with quadrupole ion trap) at m/z 1905.7. **c** N-glycan profile by endoglycosylase: GbG N-glycan chromatogram of MALDI (Matrix-assisted laser desorption/ionization) MS from m/z from 800 to 2200

N-glycan was refined from cricket (*G. bimaculatus*) origin insect glycosaminoglycan. The concentration of sodium chloride used in the ion exchange chromatography process for elution was 0 M (Fig. 1a). By confirming whether it was the low molecular weight or the degree of purity to confirm performed SAX (strong anion exchange process, Phenomex, 250 × 10 mm) - HPLC and 232 nm after glycan lyase (the heparinase I,II,III and the chondroitin ABCase) processing amid the single Peak was detected or purity was confirmed in Fig. 1a. SAX-HPLC result showed that the final low molecular weight chromatogram of cricket glycosaminoglycan noted from the major SAX-HPLC peak glycosaminoglycan of insect existed as one. After pooling this single peak followed by heparinase II treatment, heparin disaccharide IV-H and I-S standard fragments were detected in this peak using MADI-TOF mass data analysis program.

### Identification of N-glycan derived from GbG

We have already noted the monosaccharide composition of GbG [11]. N-glycans of CbG were identified as Hex_6_, Hex_5_GlcNAc_2_, Hex_6_GlcNAc_2_, Hex_7 ~ 10_GlcNAc_2_ and m/z 1905.7 of Hex_9_GlcNAc_2_ by mass/mass spectroscopy (Fig. 1b and c). We found that the neutral mono-sugar of N-glycan derived from GbG was mainly hexose [L(+) rhamnose 81.10%, D(−) ribose 7.16%, D(+) arabinose 5.91%, D(−)fructose 1.91% and D(+)glucose 1.62%] by using TMS Gas chromatography-Mass data base.

### In vivo db mouse study

#### Body weight and abdominal fat detection

**Fig. 2:**
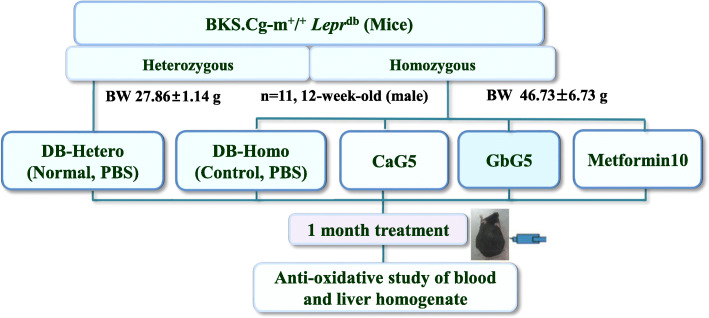
Animal experimental design: Scheme of Db mice treated with CaG or GbG over 1 month. CaG5: dung beetle (*C. molossus*) glycosaminoglycan 5 mg/kg; GbG5: field cricket (*G. bimaculatus*) glycosaminoglycan 5 mg/kg, and Metformin10: Metformin 10 mg/kg

**Fig. 3:**
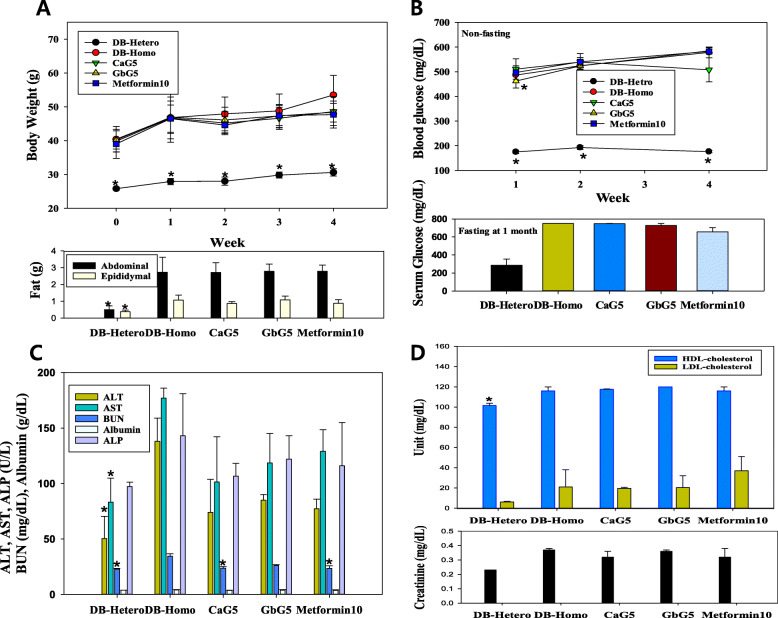
Effect of **a** Body weight, abdominal fat and epididymal fat, **b** blood glucose level, **c** Serological level of ALT, AST, ALP, BUN and albumin level, **d** HDL-cholesterol, LDL-cholesterol and creatinine levels in db mice treated with *G. bimaculatus* glycosaminoglycan for a 1 month. *p < 0.05, compared with CON (DB-Homo, diabetic) group

There were significant differences (DB-Hetero vs. DB-Homo, *p* < 0.01) in mean body weight between the normal (DB-Hetero) and all diabetic mice of treatment groups (Fig. 2) including DB-Homo during one-month of treatment (Fig. 3a). However, there were no significantly differences in total mean body weight between diabetic control (DB-Homo) and treatment groups at 1 week after beginning treatment. Mean body weights at the end of 4 weeks of treatment were as follows: DB-Hetero, 30.6 g; DB-Homo, 53.5 g; CaG5, 48.6 g (CaG5 vs. CON, *p* < 0.05); GbG5, 48.4 g; and Metformin10, 47.7 g (Metformin10 vs. CON, *p* < 0.05) (Fig. 3a). Abdominal fat weight of the treated GAG group over 1 month showed significant difference from that of the DB-Hetero (normal db mice) group, but not from that of the DB-Homo group (Fig. 3a).

#### Effects of GbG5 on blood glucose level

Glucose levels in homozygotes db mice were found to be increased with aging (from 486.5 mg/dL at the age of 12 weeks to 578.2 mg/dL at the age of 17 weeks).

Early GbG treatment in the first week lowered blood glucose level in db mice (Fig. 3b), whereas non-fasting blood glucose level in the fourth week of GbG treatment remained unchanged and no lower than at other weeks. Blood glucose levels analyzed during the first week compared with levels in the fourth week in different treatment groups via Tukey’s test, showed a statistically significant difference (*p* = 0.039).

As a positive control, low-dose metformin was used, which did not effectively reduce the non-fasting blood glucose levels compared with other GAG. Meanwhile, the fasting serum glucose levels on the last day of 1-month treatment were slightly decreased in the auto-analyzer analysis (Fig. 3b). Each treatment group compared with DB-Hetero showed a statistically significant difference (*P* < 0.23) in Tukey’s test.

#### Sero-biochemical effects after treatment with GbG

Serum albumin, alkaline phosphatase (ALP), ALT (GPT) and AST (GOT) levels in CaG- and GbG- treated groups were lower than those in the control group at 1 month after treatment, although these decreases were not statistically significant (Fig. 3c). BUN mean levels (mg/dL) in GbG-treatment db mice were lower than those in the control group. Creatinine mean levels (mg/dL) were also slightly decreased by GbG treatment (Fig. 3d). HDL-, LDL-cholesterol (Fig. 3d), and other sero-parameters (hyaluronic acid, calcium, creatinine kinase, free fatty acid, c-reactive protein, total protein, C-peptide and insulin (data not shown) detected in GbG treated group did not show any significant difference compared to those of CON.

#### Reduction of oxidative damages

**Fig. 4:**
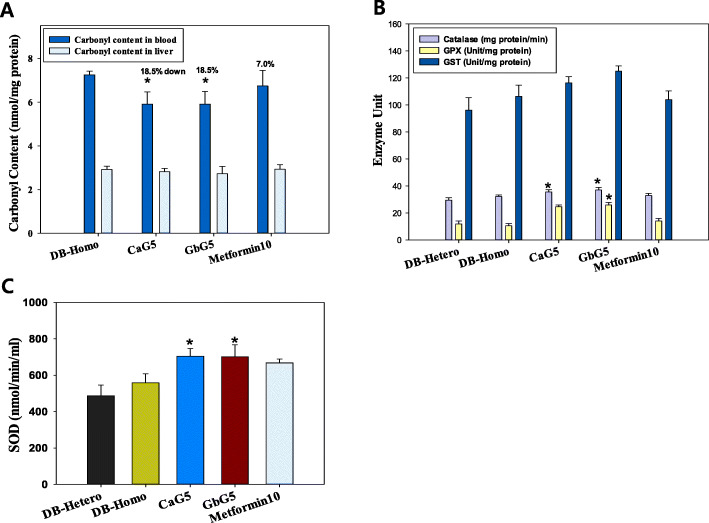
**a** Anti-oxidative effect of CaG or GbG on (**a**) carbonyl content, **b** catalase content, glutathione peroxidase (GPX), or glutathione-s transferase (GST) and **c** superoxide (SOD) content. Each value represents mean ± S.D. statistically significant from DB-Homo group (*p < 0.05)

Protein carbonyl concentrations in blood samples were significantly decreased after treatment with insect glycosaminoglycans. Decreased mean carbonyl content levels (nmol/mg protein) were: DB-Homo, 7.25; CaG5, 5.91 (CaG5 vs. CON, *p* < 0.05); GbG5, 5.91 (GbG5 vs. CON, *p* < 0.05); and Metformin10, 6.74 (Fig. 4a). However, there were no statically significant differences in hepatocyte carbonyl contents between control and treatment groups of db mice (Fig. 4a).

#### Increase of oxidative enzyme activities

As shown in Fig. 4b, levels of catalase, GPx and GST were increased in db mice treated with GbG for a month. Mean catalase activities (mg protein/min) in db mice liver after 1 month of GAG treatment were significantly increased in treatment groups: CaG5 (CaG5/CON: 110.6%, CaG5 vs. CON, *p* < 0.05); GbG5 (GbG5/CON: 114.9%, GbG5 vs. CON, *p* < 0.05); and Metformin10 (Met/CON: 102.2%). Catalase activities in GAG-treated liver groups were all increased compared to those in the control group. Mean glutathione peroxidase activities (Unit/mg protein) in all GAG treated groups were also increased in db mice: CaG5 (CaG5/CON: 235.7%); GbG5 (GbG5/CON: 248.0%, GbG5 vs. CON, *p* < 0.05) and Metformin10 (Met/CON: 135.1%). Mean activity (nmol/min/ml) ratios of glutathione-s-transferase as a detoxifying hepatic enzyme, were ensued compared with control as follows: CaG5 (109.3%); GbG5, (117.5%); and Metformin10 (97.6%). Mean activities (nmol/min/ml) of superoxide dismutase as a strong free radical scavenger were significantly increased in treatment groups: CaG5 (126.1%, CaG5 vs. CON, *p* < 0.05) and GbG5 (125.7%, GbG5 vs. CON, *p* < 0.05) (Fig. 4c).

#### Adipocyte density and pathological observations

**Fig. 5:**
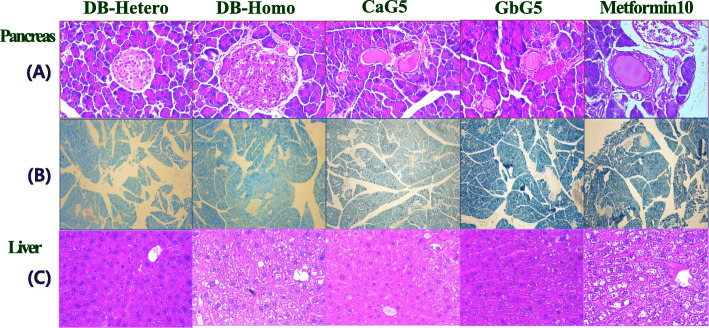
Microscopy observation of pancreas (× 400), **a** hematoxylin & eosin stained cells, **b** toluidine O-Blue stained deposits: adipocyte and **c** liver (× 50), hematoxylin & eosin stained cells in db mice treated with some GAG, (*n* = 3 per group)

Pancreas tissues were attenuated gradually diabetic damaged lesion after treatment with these GAG or metformin compared to those of non-treated Homo-db mice based on hematoxylin and eosin staining results. In general, the adipocyte cell density was counted using toluidine blue stained deposits (Fig. 5a).

Adipocyte density (cells/mm^2^) was decreased in treated pulmonary, liver and kidney tissues compared to that in the control group based on toluidine blue O staining. However, in other organ, pancreatic tissues, toluidine stain deposit number was not significantly decreased in treated group as shown in Fig. 5b. Histologically, the diabetic control group (DB-Homo) showed evidence of severe damage characterized by transformation of borders, vacuolization and cytoplasmic degranulation compared to the normal control group (DB-Hetero) of pancreatic islet (Fig. 5a). Damaged pancreas tissues with diabetic damaged lesions were reduced gradually after these GAG or metformin treatment based on the hematoxylin and eosin staining results. Administration of CaG5 or GbG5 ameliorated these damaged pancreatic islets up to 25%. According to liver tissue photograms from CON, DB-Homo, CaG5, GbG5 and metformin10 samples, the morphology of liver tissues treated with GbG5 showed the liver to be in an ameliorated diabetic, damaged state (Fig. 5c).

### In vitro D-HMVEC cell study

#### The role of endothelial nitric oxide synthase, laminin and VEGF on HUVEC

**Fig. 6:**
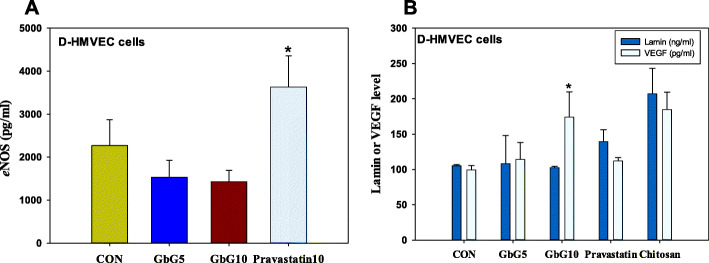
Effect of **a** endothelial nitric oxide synthase (*e*NOS), **b** laminin and vascular endothelial growth factor (VEGF) on human microvascular endothelial cells from type 2 diabetics. As a D- HMVEC cell study, GbG5: *G. bimaculatus* glycosaminoglycan 5 mg/ml; GbG10: *G. bimaculatus* glycosaminoglycan 10 mg/ml; Pravastatin: pravastatin 10 mg/ml and Chitosan: chitosan 10 mg/ml. Each value represents mean ± S.D. statistically significant from DB-Homo (*p < 0.05)

Endogenous nitric oxide (*e*NOS, pg/ml) levels in human cardiac microvascular endothelia cells (from diabetic type 2) were decreased after treatment with 5 or 10 mg/ml of GbG5 (67.4%) or GbG10 (62.9%). However, after treatment with 10 mg/ml pravastatin, *e*NOS (pg/ml) was significantly increased: Pravastatin10 (160.16%, Pravastatin10 vs. CON, *p* < 0.05) (Fig. 6a). Laminin level (ng/ml) in the same cell type was not significantly changed after treatment with either GbG or positive control: GbG5 (102.9%); GbG10 (97.6%); Pravastatin (132.7%) and chitosan (196.9%). VEGF levels (*p*g/ml) in HMVEC diabetic cells showed dose-dependent increases after GbG treatment: GbG5 (114.7%); GbG10 (175.1%, GbG10 vs. CON, *p* < 0.05); Pravastatin10 (112.7%) and chitosan10 (185.8%) (Fig. 6b).

## Discussion

Glycosaminoglycan treatment effectively decreased blood glucose levels in diseases associated with hyperglycemia [24]. Glycosaminoglycan, extracted from the mucous membrane of crickets’ inner cortex, has been reported to exhibit anti-lipidemic effect on rats with high-fat diet-induced diabetes [11]. It also lowered the blood pressure [25] and induced anti-inflammatory effects in rats with adjuvant-induced edema [9]. Results of our db mouse study show that after treatment with each glycosaminoglycan, the serum BUN levels decreased (CaG5, 68.5%; GbG5, 75.5% and Metformin10, 68.4%) compared with the levels in the DB-Homo group. Furthermore, the total levels of albumin, alkaline phosphatase, ALT, AST and creatinine were also decreased by GAGs, which ameliorated metabolic activation of hepatocytes. The oxidative enzymes expressed serum biochemical biomarkers including albumin, ALP, ALT, AST and creatinine. Serum insulin level was not significantly altered between control and treated groups. The db mouse model of type II diabetes is not primarily dependent on blood glucose control hormone and insulin secretion but is affected by β-cell dysfunction and insulin resistance. Hence, when we analysed the serum C-peptide and insulin levels between the insect GAG-treated groups and the db untreated (CON) group, no difference was seen in the levels of C-peptide (< 0.1 ng/mL) and insulin (< 0.2 μU/mL) between groups according to Green-Cross Lab Cell analysis. Oral glucose tolerance tests in diabetic mice using these insect GAGs are necessary to ascertain their anti-diabetic effects even though they were not accurate because the orally administered GAGs are also digested and increase the levels of mono (neutral, amino and acid) sugars.

In fact, specific functional groups including epoxy, hydroxyl and carbonyl contribute to potent free radical-scavenging activity [26]. In addition to anti-oxidant activities, GbG exhibits unremarkable anti-diabetic activity without adverse effects in in vitro or in vivo models.

Glycosaminoglycan also acts as an anti-oxidant by scavenging free radicals that induce cellular oxidative damage [27]. However, the effect of enzymes such as SOD, along with cofactors Cu or Zn, on cardiovascular target sites results in increased anti-oxidant defence [28]. Levels of anti-oxidative enzymes and activities of catalase, glutathione peroxidase, glutathione-s-transferase and SOD were also increased by GbG. Thus, hepatocellular oxidative stress triggered by free radical damage is attenuated by these anti-oxidant enzymes. In our db mice experiment, GbG5 appeared to act as an antioxidant. It increased catalase activity by 114.9%, GPX by 248.1%, GST by 117.6% and SOD by 125.7%. In terms of the blood cellular oxidative damage, protein oxidative damage was also reduced (CaG5 by 18.5%; GbG5 by 18.5% and Metformin10 by 7.0%) by these GAGs based on blood neutrophil carbonyl content.

However, as shown in Fig. 3d, increased HDL cholesterol protects blood vessels and LDL cholesterol reduction is a positive outcome. However, from the perspective of a human clinical study, we recognise that a GAG-lyase deficient patient is an exception in that the GAGs and mucopolysaccharides may persist in the blood.

**Fig. 7:**
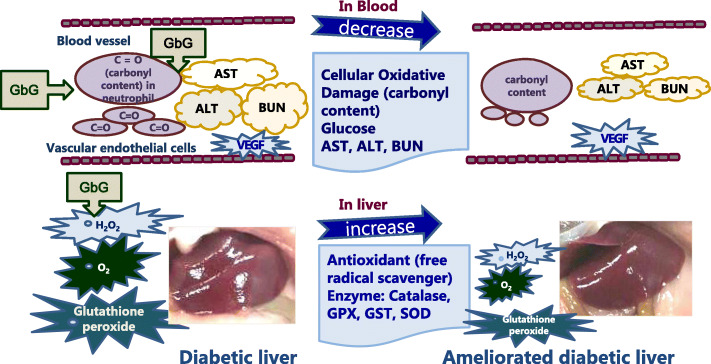
The proposed mechanism of action on cricket (*G. bimaculatus*) glycosaminoglycan in db mice: antioxidative effect of GbG glycosaminoglycan on db mice

Purified human GAGs have been shown to reduce cell death, limit DNA fragmentation and thus protein oxidation, decrease the level of OH· generation and lactate dehydrogenase activity, inhibit lipid peroxidation and thereby improve endogenous antioxidant defences [29]. However, mucopolysaccharidosis type II (MPS II), a lysosomal storage disorder caused by deficient enzyme iduronate-2-sulphatase that is responsible for the degradation of glycosaminoglycans dermatan and heparin sulphate, is protected against lipid peroxidation while protein damage in these patients is repaired by enzyme replacement therapy [30]. The proposed mechanism of action of cricket glycosaminoglycan in db mice is summarised in Fig. 7. The postulated anti-oxidative action of cricket glycosaminoglycan in type 2 diabetic mice is supported by findings suggesting that GAG such as the low-molecular-weight heparin, increases SOD levels and inhibits oxidative stress [31] and that glycosaminoglycan derived from *Urechis unicinctus* significantly enhances liver SOD and GSH-Px activities [24]. Further clinical studies are needed to verify the proposed mechanism. Heparinoid (a type of glycosaminoglycan) sources from animals and fish may be associated with viral infection in addition to endangering marine life such as whales and sharks. Therefore, an in vitro insect rearing system provides a large quantity with the same quality control.

## Conclusions

The results based on serum biochemical and hepatocellular antioxidant assays in db mice suggest that cricket (*G. bimaculatus*) glycosaminoglycan can be used as a natural antioxidant agent and functional food for the treatment of diabetes.

## Data Availability

All data generated or analyzed during this study are indicated in this article (with no patient data). The datasets generated during and /or analyzed during the current study are available from the corresponding author on reasonable request.

## Abbreviations

ALP: Alkaline phosphatase
ALT (GPT): Glutamate pyruvate transaminase
AST (GOT): Glutamate oxaloacetate transaminase
BUN: Blood urea nitrogen
CaG: Dung beetle (*C. molossus*) glycosaminoglycan
CaG5: *C. molossus* glycosaminoglycan 5 mg/kg
CON: Control group
GAG: Glycosaminoglycan
DB(db): Diabetic
DB-Hetero: Heterozygotes db normal mice
DB-Homo: Homozygotes diabetic mice
D-HMVEC: Diabetic type2 microvascular endothelial cell
GbG: *G. bimaculatus* (a type of cricket) glycosaminoglycan
GbG5: *G. bimaculatus* glycosaminoglycan 5 mg/kg
GC-MS: Gas chromatography with mass detector
GPx: Glutathione peroxidase
GST: Glutathione s-transferase
HDL: High-density lipoprotein
LDL: Low-density lipoprotein
Metformin10: Metformin 10 mg/kg
*e*NOS: Endothelial nitric oxide synthase
SOD: Superoxide dismutase
VEGF: Vascular endothelial growth factor
TMS: Trimethylchlorosilane
